# MG53 suppresses tumor progression and stress granule formation by modulating G3BP2 activity in non-small cell lung cancer

**DOI:** 10.1186/s12943-021-01418-3

**Published:** 2021-09-14

**Authors:** Haichang Li, Pei-Hui Lin, Pranav Gupta, Xiangguang Li, Serena Li Zhao, Xinyu Zhou, Zhongguang Li, Shengcai Wei, Li Xu, Renzhi Han, Jing Lu, Tao Tan, Dong-Hua Yang, Zhe-Sheng Chen, Timothy M. Pawlik, Robert E. Merritt, Jianjie Ma

**Affiliations:** 1 Department of Surgery, The Ohio State University College of Medicine, Columbus, OH 43210 USA; 2 Department of Pharmaceutical Sciences, College of Pharmacy and Health Sciences, St. John’s University, Queens, NY 11439 USA

**Keywords:** MG53, G3BP2, Cisplatin, Stress granules (SGs), Non-small cell lung cancer (NSCLC)

## Abstract

**Background:**

Cancer cells develop resistance to chemotherapeutic intervention by excessive formation of stress granules (SGs), which are modulated by an oncogenic protein G3BP2. Selective control of G3BP2/SG signaling is a potential means to treat non-small cell lung cancer (NSCLC).

**Methods:**

Co-immunoprecipitation was conducted to identify the interaction of MG53 and G3BP2. Immunohistochemistry and live cell imaging were performed to visualize the subcellular expression or co-localization. We used shRNA to knock-down the expression MG53 or G3BP2 to test the cell migration and colony formation. The expression level of MG53 and G3BP2 in human NSCLC tissues was tested by western blot analysis. The ATO-induced oxidative stress model was used to examine the effect of rhMG53 on SG formation. Moue NSCLC allograft experiments were performed on wild type and transgenic mice with either knockout of MG53, or overexpression of MG53. Human NSCLC xenograft model in mice was used to evaluate the effect of MG53 overexpression on tumorigenesis.

**Results:**

We show that MG53, a member of the TRIM protein family (TRIM72), modulates G3BP2 activity to control lung cancer progression. Loss of MG53 results in the progressive development of lung cancer in *mg53*^*-/-*^ mice. Transgenic mice with sustained elevation of MG53 in the bloodstream demonstrate reduced tumor growth following allograft transplantation of mouse NSCLC cells. Biochemical assay reveals physical interaction between G3BP2 and MG53 through the TRIM domain of MG53. Knockdown of MG53 enhances proliferation and migration of NSCLC cells, whereas reduced tumorigenicity is seen in NSCLC cells with knockdown of G3BP2 expression. The recombinant human MG53 (rhMG53) protein can enter the NSCLC cells to induce nuclear translation of G3BP2 and block arsenic trioxide-induced SG formation. The anti-proliferative effect of rhMG53 on NSCLC cells was abolished with knockout of G3BP2. rhMG53 can enhance sensitivity of NSCLC cells to undergo cell death upon treatment with cisplatin. Tailored induction of MG53 expression in NSCLC cells suppresses lung cancer growth via reduced SG formation in a xenograft model.

**Conclusion:**

Overall, these findings support the notion that MG53 functions as a tumor suppressor by targeting G3BP2/SG activity in NSCLCs.

**Supplementary Information:**

The online version contains supplementary material available at 10.1186/s12943-021-01418-3.

## Background

Lung cancer is a leading cause of mortality worldwide, afflicting approximately 170,000 people each year in the United States, and non-small cell lung cancer (NSCLC) accounts for approximately 80% of all lung cancer cases [1, 2]. In 2020, it is estimated that the United States will have more than 1806,590 new cancer cases, and lung cancer accounted for more cancer-related deaths than breast, prostate, and colon cases combined [2]. Despite available surgical and chemotherapeutic treatment modalities, the prognosis associated with lung cancer remains grim. In the United States, from 2007 to 2013, overall 5-year survival was only 23.6% for NSCLC [3]. There is a great need for novel therapeutic agents to treat NSCLC more effectively. The search for novel tumor-suppressive factors that inhibit tumor progression represents an important area of cancer research.

Under stress conditions, such as hypoxia, nutrient deprivation, heat shock, or oxidative stress, cells have to save energy by silencing or reducing protein synthesis for survival. One mechanism to regulate protein synthesis is the formation of stress granules (SGs) [4–8]. These granules contain scaffold and RNA-binding proteins, which keep the mRNAs translationally silent while protecting the cells from harmful conditions [9–12]. G3BP2 is a known oncogene and a main component of SGs [13–22]. Several lines of studies have demonstrated that cancer cells develop resistance to chemotherapeutic treatment and radiotherapy by the excessive formation of SGs [23–29]. As a result, cancer cells use SGs to support survival and metastatic capacity when exposed to radiotherapy or chemotherapy. Thus, therapeutic approaches that selectively control SG formation might represent a potentially effective means to enhance the treatment of cancer [30–32]

A recent series of studies from our group identified MG53 (also known as TRIM72) as an important component of cell membrane repair [33–35]. Mice with ablation of the MG53 gene develop pathology in multiple tissues and organs due to defective cell membrane repair [33, 36–40]. Transgenic mice with increased levels of MG53 in the bloodstream (tPA-MG53, ~100-fold higher than wild type mice) live a healthy lifespan and display increased tissue-repair and regenerative capacity following injury [41].

In addition to facilitating tissue repair, MG53 contains a conserved RING domain with intrinsic E3-ligase activity [42–44] and coiled-coiled structures [45] that can interact with other signaling proteins to modulate cell proliferation and survival under stress conditions. Knockout of TRIM72/MG53 through CRISPR-gene silencing led to aggressive lung tumor growth and metastasis in mice, raising the possibility that MG53 might possess tumor suppressor function in lung cancer [46, 47]. However, the underlying mechanisms that govern MG53’s potential tumor suppression role remain largely unknown.

In this study, we uncover a new mechanism for MG53’s anti-tumor function involving the control of G3BP2/SG signaling. We performed allograft transplantation of mouse NSCLC cells into the *mg53*^*-/-*^, wild type, and tPA-MG53 mice, and found aggressive lung tumor growth in the *mg53*^*-/-*^ mice compared with wild type mice, whereas lung tumor growth was significantly suppressed in the tPA-MG53 mice. Consistent with findings from other investigators [16, 17, 21, 22, 48], we detected elevated levels of G3BP2 in tumor versus non-tumor human lung tissues. We found that the TRIM-motif of MG53 can interact with G3BP2 to regulate SG formation in NSCLC cells under stress conditions. We developed a unique model with the inducible secretion of MG53 in cancer cells and demonstrated that tailored overexpression of MG53 could impact G3BP2/SG signaling and mitigate NSCLC growth in a xenograft model.

## Materials and methods

### Animal care, in vivo tumor mouse model, and human tissue samples

All animal care and usage followed NIH guidelines and IACUC approval by The Ohio State University. *mg53*^*-/-*^ mice, *tPA-MG53* mice, and their wild type (WT) littermates were bred and generated as previously described [33, 41]. Nude mice (age 8-10 weeks) were purchased from Taconic Biosciences (*Ncr Nude*).

### Chemicals, and recombinant human MG53 protein (rhMG53)

All chemicals were obtained from Sigma-Aldrich unless otherwise described. rhMG53 protein was purified from *E. coli* fermentation as described previously [49].

### Cell culture, viability assay and stress treatment

HEK293, HEK293T, and Human non-small cell lung cancer (NSCLC) cell lines (A549, H2122, and H460) were obtained from the American Type Culture Collection (ATCC). The mouse NSCLC cell line (*Braf* ^*V600E/+*^*; Atg7*^*−/−*^*)* was kindly provided by Dr. Strohecker (OSU) [50]. The cells were grown in RPMI 1640 medium supplemented with 10% FBS, 100 U/ml penicillin, and 100 μg/ml streptomycin at 37^o^C in the presence of 5% CO_2_. For stress induction, cells were treated with 0.5 mM arsenic trioxide (ATO, Sigma Aldrich) for indicated time points. A549 cells were seeded in 96-well plates and cultured for 24 hours, then treated with varying concentrations of rhMG53 (0.05-100 μg/ml) or rhMG53 plus cisplatin (5 μM) for either 48 hours or 72 hours. Cells were detached by 0.25% Trypsin-EDTA solution, and their viability were quantified by flow cytometry (CytoFLEX S system, Beckman Coulter).

### Tumor mouse models

#### Mouse NSCLC allograft model

The mouse NSCLC cells *(Braf* ^*V600E/+*^*; Atg7*^*−/−*^*)* (at 4 x10^6^ per site) were mixed with an equal volume of Matrigel (BD Biosciences) and implanted subcutaneously to both flanks of *WT*, *mg53*^*-/-*^ or *tPA-MG53* (TPA) mice (8-10 weeks old). Mice were monitored for 4 weeks for tumor formation.

#### Human NSCLC xenograft nude mouse model

Nude mice were implanted subcutaneously in the right flank with 2 x10^6^ of either A549 cells or A549 transduced with Adenovirus (Ad-TRE-tPA-MG53), which expresses tPA-MG53 in a Doxycycline (Dox)-dependent manner. The group of mice which were implanted with A549 (Ad-TRE-tPA-MG53) was subsequently randomly divided into two subgroups; one subgroup received saline and the other subgroup received Doxycycline (Dox) (100 μg/tumor, subcutaneous injection in 100 μl saline). Mice were monitored for two months. During the experiment, mice were examined daily, and tumor length and width were measured using a caliper. Tumor size was determined using the following formula: ½ (length X width ^2^) [51]. According to the IACUC guideline, the experiments will terminate when the tumor size reaches 15 mm. At the end of the experiment, mice were sacrificed. Xenografts were removed, photographed, and quantified.

### Human lung tumor procurement

Human non-small cell lung cancer and the adjacent non-cancerous tissues were obtained through the OSUCCC Tissue Procurement Shared Resource, based on a research protocol approved by the OSU Institutional Review Board (IRB).

### Plasmids

#### HA-tagged MG53 full length and deletion mutant plasmids

HA-MG53(FL)-the sequence encoding the full length (nucleotides1-1434) human *trim72* (*mg53*) was PCR synthesized and cloned into a pHM6 expression vector (Roche) which expresses HA (hemagglutinin) tag sequence YPYDVPDYA on its N-terminal. The HA-MG53(N)- which contains the N-terminal (nucleotides1-807) encoding MG53 amino acids1-269; and the HA-MG53(C) which contains the C-terminal (nucleotides 738-1434) encoding MG53 amino acids 246-477 were PCR synthesized and similarly cloned into the pHM6 vector. All plasmids were verified by Sanger Sequencing. RFP-MG53 was described previously [33].

#### Lentiviral-shRNA constructions

The pKLO-scr shRNA (5’-GACTGACATGTCAAGCTGTAC-3’) and the pKLO-shMG53 shRNA (5′-GAAGAGTGTGGCTGTGCTGGAGCATCAG-3’) was reported previously [52]; pKLO-G3BP2 shRNA (5’-CGGGAGTTTGTGAGGCAATAT-3’) was designed and ligated into pKLO-mcherry-puro vector [53]. All plasmids were confirmed by sequencing. HEK293T cells were transfected with three plasmids listed below to produce Lenti-virus. The three plasmids pLKO –shRNA construct(s), pCMV- NTR (encoding gag, pol, and rev genes), and VSV-G (expressing envelope gene) were mixed at a ratio of 10:9:1 and transfected into HEK293T cells with Lipofectamine LTX Reagent. 24 hr post-transfection, the medium was changed to regular medium and cells were incubated for an additional 24 hr. The supernatant containing the packaged lentiviral particles was harvested 48 hr post-transfection and purified. This viral preparation was then used to infect H2122 cells. The stable Lentiviral transduced cells expressing the target shRNA were positively selected by incubation with a puromycin (10μg/ml)-containing selection medium for two weeks. The selected clone D was used as shMG53 knockdown cell line (shMG53-D, see Fig. 4A). The selected clone E was used in this study as shG3BP2 knockdown cell line (shG3BP2-E, see Fig. 4B).

#### Ad-tPA-MG53-mcherry constructions

The full-length MG53 cDNA (accession no: AB231474) was cloned into a pShuttle vector containing tet-inducible gene expression downstream of a tetracycline (*tet*)-responsive element (TRE) to generate inducible gene expression downstream Ad-tPA-MG53-mcherry plasmid. Adenoviral vector AdtPA-MG53-mcherry was transfected into HEK293T cells using Lipofectamine LTX reagents (Invitrogen) per the manufacturer’s recommendations. Viral particles were produced according to a previous study [53].

### Transwell cell invasion assay

Cells (1×10^5^) were resuspended in serum-free DMDE medium and added to 24-well format of Transwell chambers (Corning) precoated with matrigel (BD Bioscience). Serum was added to the bottom wells of the chambers to induce cell migration. After 24 hr, the cells that had migrated through the membrane were fixed with 10% formalin for 10 min. and stained with hematoxylin for 30 min. Wells were washed and the non-invading cells were removed from the upper surface of the membrane. The invading cells were counted in five random fields and expressed as % of the average number of cells/fields under a microscope.

### Colony formation assay

Anchorage-independent growth assays in soft agar were performed as described [54]. Briefly, the 1.5 % bottom layer of Seaprep agar (Lonza) containing 4% FBS in 1X RPMI was first plated into 35 mm-well plates. Suspension of 5000 cells was assayed per well in 1.2 % Seaprep top agar layer. Cells were fed every 7 days. At the end of the assay, cultures were stained with 0.5 ml crystal violet for 1 hr, washed, and imaged.

### MTT cell proliferation assay

For evaluating the effect of rhMG53 on the proliferation of NSCLC cells, MTT assay was used. Cells were plated at 2,000 cells per well in a 96-well plate and then treated with indicated amounts of rhMG53 for 72 hr. 20 μl MTT [3-(4,5-dimethylthiazol-2-yl)-2,5-diphenyltetrazolium bromide] (5 mg/mL in PBS) was added to each well and further incubated for 4 hr. Absorbance was recorded at 570 nm after adding 150 μL DMSO.

### Antibodies, western blotting, and co-immunoprecipitation analysis

Primary antibodies used in this study are as follows: anti-PABP-1 and anti-tubulin (Cell Signaling Technologies), anti-Ki67 (Sigma), anti-G3BP2 (Novus, Cat #NBP1-82976) [8] and anti-Lamin A/C (Abcam), and anti-G3BP1, anti- β-actin, anti-GAPDH (Santa Cruz Biotechnology), Anti-HA (Biolegend). Anti-MG53 is a custom-made rabbit monoclonal antibody.

The cytoplasmic and nuclear protein extraction were performed by NE-PER^TM^ Nuclear and Cytoplasmic Extraction kit (Thermo Fisher Scientific) according to the manufacturer’s instructions. Cell and tissue lysates were used for immunoblotting, as previously described [38]. Briefly, after blocking, membranes were incubated with relevant antibodies and probed with corresponding HRP-conjugated secondary antibodies (Jackson Immunoresearch). All films were developed with ECL-Plus regents (GE healthcare) and imaged using ChemiDoc^TM^ Gel Imaging System (Bio-Rad).

For Co-immunoprecipitation (co-IP): HEK293 cells were transfected with HA-MG53 (full length or deletion mutants) and Myc-DDK-tagged G3BP2 (Origene) for 24 hr and incubated in media containing proteasome inhibitor MG132 (10 μM, Selleck Chemicals) for 16 hr. Cells were re-suspended in RIPA buffer plus protease inhibitor cocktail (Sigma) and pre-cleared with a mixture of protein-A and protein-G beads (KPL Inc.). Immunoprecipitation was performed with anti-HA magnetic beads (ThermoFisher). The resulting immuno-complexes were collected and separated by SDS-PAGE.

### Confocal microscopy

Confocal images were taken with Zeiss 780 or Nikon A1R confocal microscope. The images were analyzed using ImageJ software.

### Histology and immunofluorescent staining

Histology and immunofluorescent staining were performed as previously described [38, 39]. Briefly, tissues were dissected and fixed in 4% Paraformaldehyde (PFA) overnight at 4°C. After fixing, samples were washed three times for 5 min with 70% ethanol. Washed samples were processed and embedded in paraffin. 4-μm thick paraffin sections were cut. Cells were fixed with 4% PFA.

### Statistical analysis

All data are expressed as means ± SD. For each experiment, three independent replicates were performed. Statistical evaluation was conducted using Student t-tests and by ANOVA for repeated measures (Graphpad Prism 8.2). A value of P<0.05 was considered statistically significant.

## Results

### Loss of MG53 promotes lung tumorigenesis in mg53^-/-^ mice

**Fig. 1:**
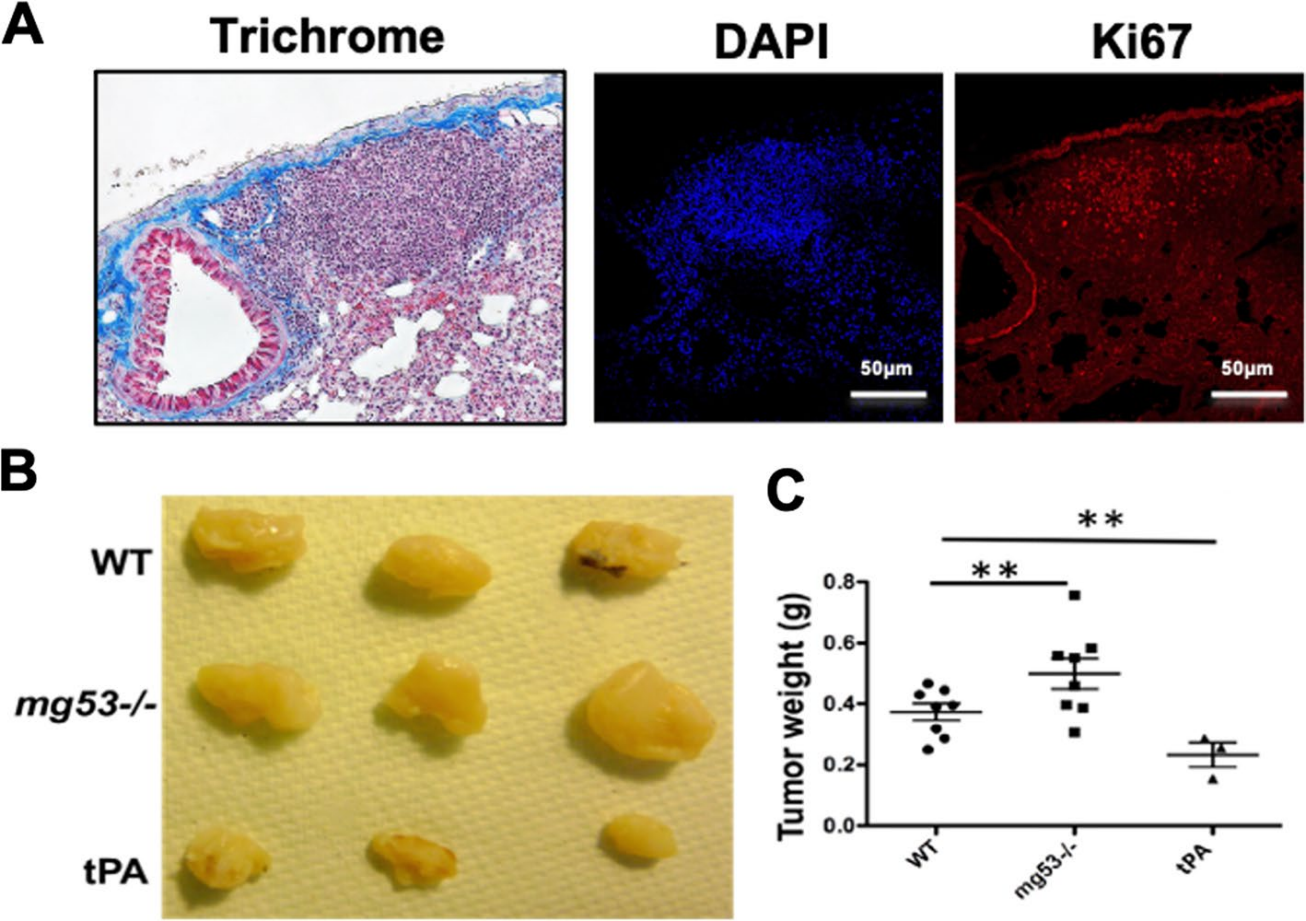
Higher frequency of lung tumorigenesis in aged *mg53*^*-/-*^ mice. **A** IHC and pathological analysis revealing the presence of abnormal cell proliferation in the lung tissue derived from aged *mg53*^*-/-*^ mice (10-months or older) (left panel, Masson Trichrome stain); DAPI indicates nucleus and anti- Ki67 serves as a proliferation marker for human tumor (right). A total of 8 (8/20) lung tissues from *mg53*^*-/-*^ were observed with abnormal cell proliferation. **B** Tumor growth assay was examined in *mg53*^*-/-*^*,* tPA-MG53, and WT mice as described in materials and methods. After 4 weeks, the primary tumors were excised, weighted, and photographed. **C** Quantification of tumor weight from *mg53*^*-/-*^, tPA-MG53, and WT mice. Each dot represents an individual mouse, with a line demarcating the median for each cohort with S.D. ** *p*<0.01

Studying *mg53*^*-/-*^ mice, we noticed an interesting phenomenon: *mg53*^*-/-*^ mice often develop lung tumors as they age. As shown in Fig. 1A, histological staining revealed localized areas in the *mg53*^*-/-*^ lung sections with aggressive tumor proliferation, which were positive for staining using the solid tumor marker Ki67 [55]. Approximately 55% of *mg53*^*-/-*^ mice (10-months or older, 10 out of 18 mice in both genders) showed high Ki67-staining in the lung sections, whereas only 11% (2 out of 18) age-matched wild type mice demonstrated such aggressive proliferation pattern.

To understand the physiologic role of MG53 in regulation of tumor growth, we conducted allograft transplantation of mouse NSCLC cells (*Braf* ^*V600E/+*^*Atg7*^*−/−*^*)* ^50^ into wild type, *mg53*^*-/-*^, and tPA-MG53 mice [41]. 4x10^6^ cells were injected into the flanks of mice subcutaneously. At 4 weeks after transplantation, solid tumors were explanted and weighed (Fig. 1B). Compared with wild type mice, significantly larger solid tumors were found in the *mg53*^*-/-*^ mice (Fig. 1C). In contrast, smaller solid tumors were formed in the tPA-MG53 mice, which contain elevated levels of MG53 in the bloodstream. These findings substantiate the notion that MG53 has a tumor suppressor function in lung cancer [46, 47].

### Human NSCLCs show elevated G3BP2 expression and are predominantly localized in cytosol

**Fig. 2:**
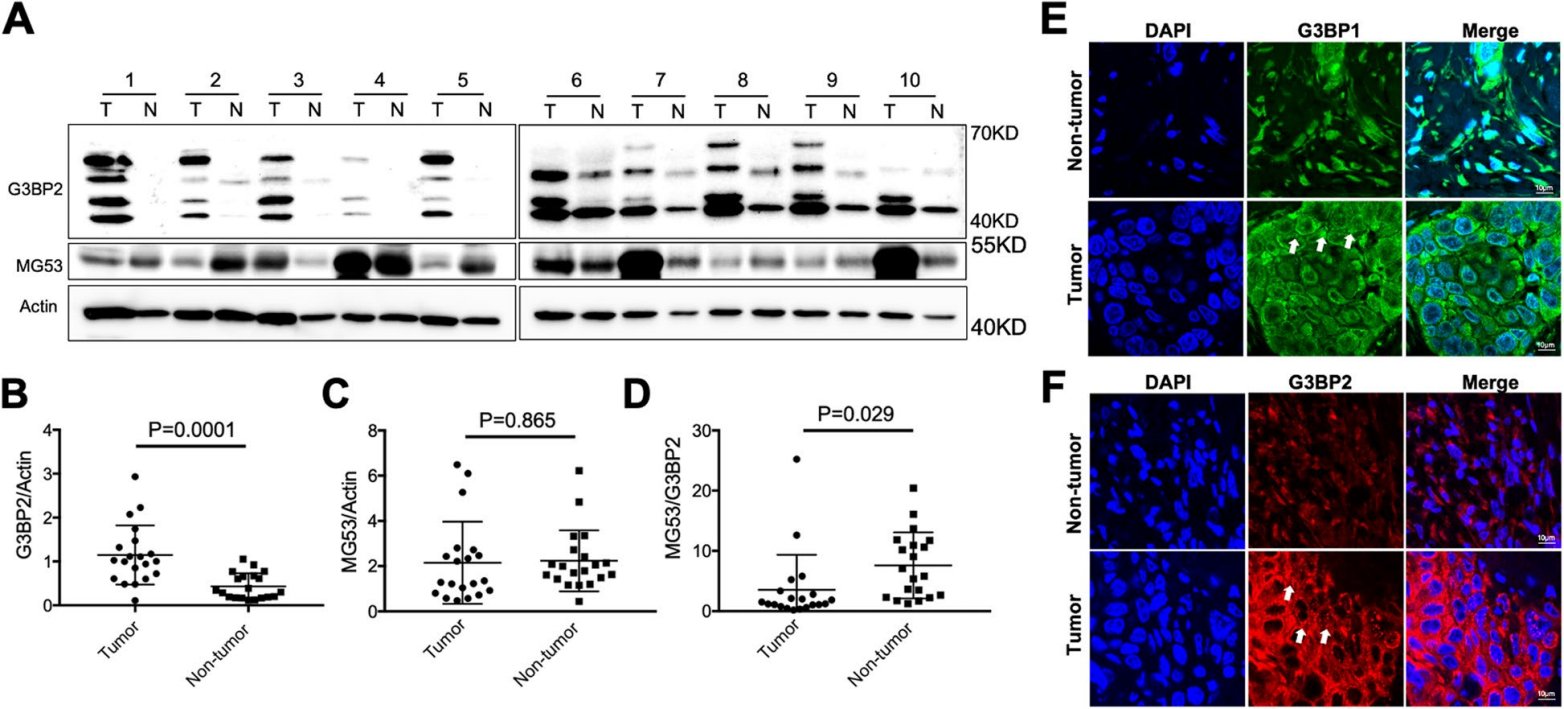
Elevated G3BP2 expression was detected in Human NSCLC. Expression of MG53 and G3BP2 from the human NSCLC tumor tissues (**T**) and their adjacent non-tumor lung tissues (**N**) were subjected to western blotting analysis. **A** Representative Western blot with anti-G3BP2 and Anti-MG53 antibodies, actin as loading control. Quantification of the relative expression levels of G3BP2 (**B**) MG53 (**C**) and MG53/G3BP2. **D** among lung tumors and the adjacent non-tumor tissues (*n*=20). **E** Representative IHC staining of G3BP1 (SG marker) on human NSCLC lung samples. **F** Representative IHC staining of G3BP2 on human NSCLC lung samples. The nucleus was stained with DAPI (blue). Arrows indicate the SG formation

Through collaboration with the Tissue Repository Center at The Ohio State University, we obtained 20 human NSCLC patient samples from tumor and adjacent non-tumor lung tissues. Western blot showed that G3BP2 protein was up-regulated in tumor (T) versus non-tumor (N) samples (Fig. 2A, see Supplemental Figure S1 for additional human lung tissue samples). We normalized the level of G3BP2 protein over actin as the loading control and found significant elevation of G3BP2 in the lung tumor (Fig. 2B, *p*=0.0001). We found that MG53 showed various expression levels in the different patient samples (which may reflect the different injury status of the lung tissue), but on average, there were no significant differences between tumor and non-tumor samples (*p*=0.865) (Fig. 2C). Interestingly, we found that the ratio of MG53/G3BP2 is significantly reduced in tumor compared to non-tumor samples (Fig. 2D, *n*=20).

We conducted immunohistochemical (IHC) staining using antibodies against G3BP1 and G3BP2 with the paired lung sections derived from the tumor (Fig. 2E, *bottom*) and non-tumor regions (Fig. 2E, *top*). G3BP1 has been widely used as a biomarker of SG [14, 15]. As shown in Fig. 2E, the intensity of G3BP1 staining was higher in tumors compared with non-tumor lung sections. Western blot confirmed the elevation of G3BP1 protein in the human lung tumors (Supplementary Figure S1), suggesting elevated SG is a pathological feature of NSCLC. High intensity G3BP2 staining was observed in the tumor region compared with non-tumor region, with distinct puncta structure in the cytosol (Fig. 2F, *arrow marks*). Similar observations were also made in lung sections derived from *mg53*^*-/-*^ mice (10 months age), which contained more cytosolic granule structures than those from the wild type mice (Supplemental Figure S2). The abundant cytosolic distribution of G3BP2 is consistent with its role in the cytosolic SG formation in human cancer patients [17, 19, 22, 56].

### The amino terminus of MG53 interacts with G3BP2

**Fig. 3:**
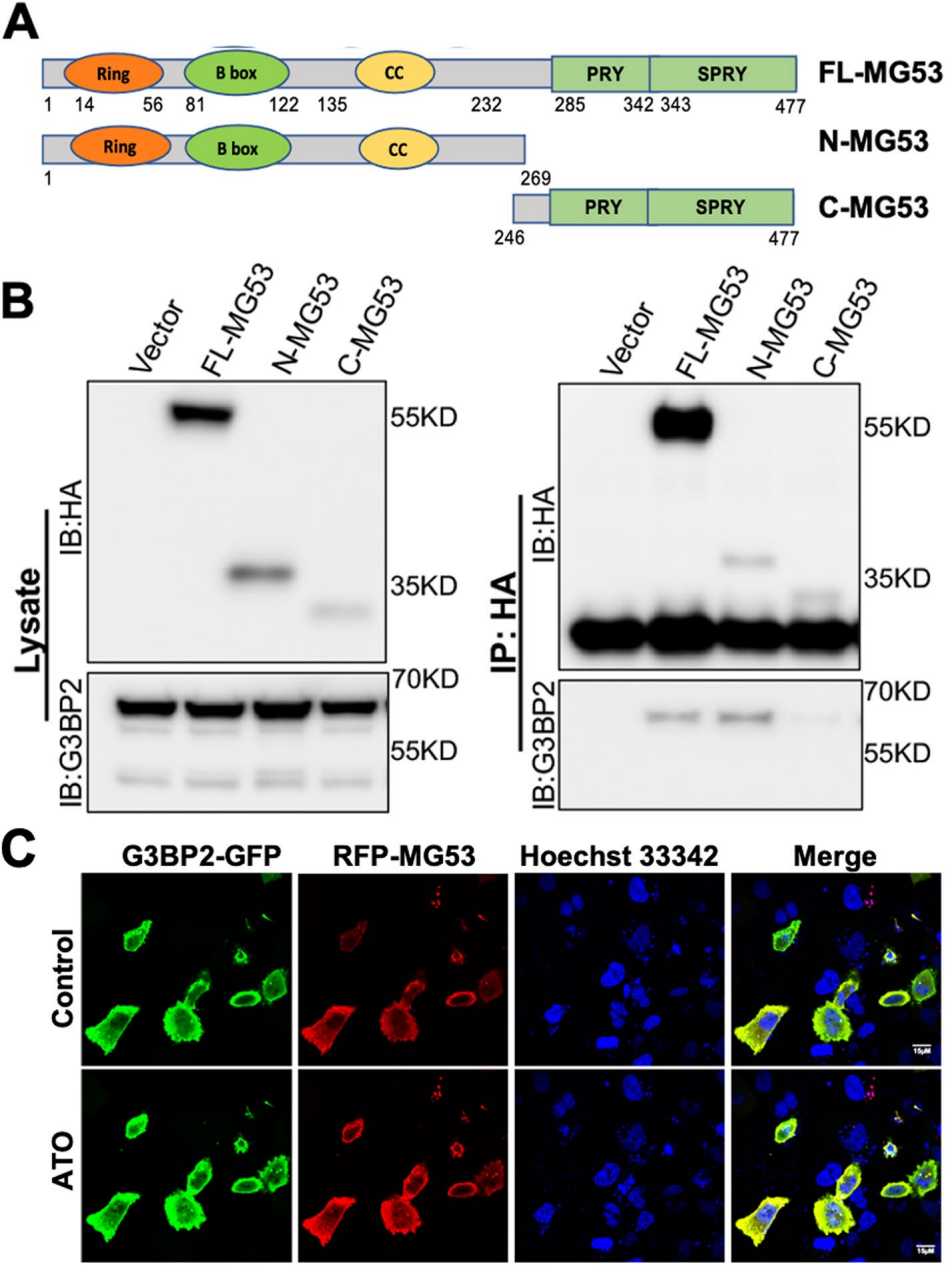
TRIM domain of MG53 interacts with G3BP2 in NSCLC cells. **A** Schematic representation of the domain structures and the encoding amino acid numberings of MG53 (FL) and its N-terminal or C-terminal construct. **B** HEK293 cells were transfected with FL-MG53, N-MG53, and C-MG53 for 24 hr. The whole cell lysate was subjected to western blot assay using anti-HA and anti-G3BP2 (left). The immunoprecipitation (IP) assay using anti-HA-conjugated magnetic beads, then performed with anti-HA (right, top panel) and anti-G3BP2 (right, bottom panel). **C** A549 cells were transfected with RFP-MG53 and G3BP2-GFP for 24 hr. The nucleus (blue) was labeled with Hoechst 33342. The co-localization of MG53 and G3BP2 was examined under confocal microscopy (*top*). ATO treatment induces nuclear translocation of both RFP-MG53 and G2BP2-GFP (*bottom*)

Co-immunoprecipitation (co-IP) studies demonstrated a physical interaction between MG53 and G3BP2 (Fig. 3). Human MG53 protein contains an amino-terminal tripartite motif (TRIM) including a RING domain, a B-box domain, and coiled-coil domain [42–44, 57]. We generated deletion variants of MG53: the full-length MG53 construct (FL-MG53, aa 1-477), N-MG53 (aa 1-269) containing TRIM domain, and C-MG53 (aa 246-477) containing the PRY-SPRY domains (Fig. 3A). For co-IP and western blot purposes, a hemagglutinin (HA) tag was added to the amino terminal end of the different MG53 constructs, and transiently expressed in HEK293 cells. As shown in Fig. 3B, co-IP identified physical interactions between G3BP2, FL-MG53, and N-MG53, whereas the C-MG53 could not pull-down G3BP2. These findings reveal that the TRIM domain of MG53 likely contains the active motif to facilitate interaction with G3BP2.

We constructed RFP-MG53 (human MG53 sequence) and G3BP2-GFP fluorescent constructs, which allowed us to follow the subcellular distribution of MG53 and G3BP2 under live-cell confocal imaging. Clearly, there was significant co-localization between RFP-MG53 and G3BP2-GFP in A549 cells under basal condition (Fig. 3C, *top panels*). Interestingly, we found that cells treated with arsenite trioxide (ATO, 0.5 mM, see also Fig. 5) displayed nuclear translocation of RFP-MG53 and G3BP2-GFP (Fig. 3C, *bottom panels*). Supplemental Movie illustrates the dynamic co-movements of RFP-MG53 and G3BP2-GFP in A549 cells following ATP treatment. The implication of the nuclear translocation of MG53/G3BP2 will be examined in Fig. 5.

### Anti-proliferative effect of MG53 in human NSCLC cells is mediated by G3BP2

**Fig. 4:**
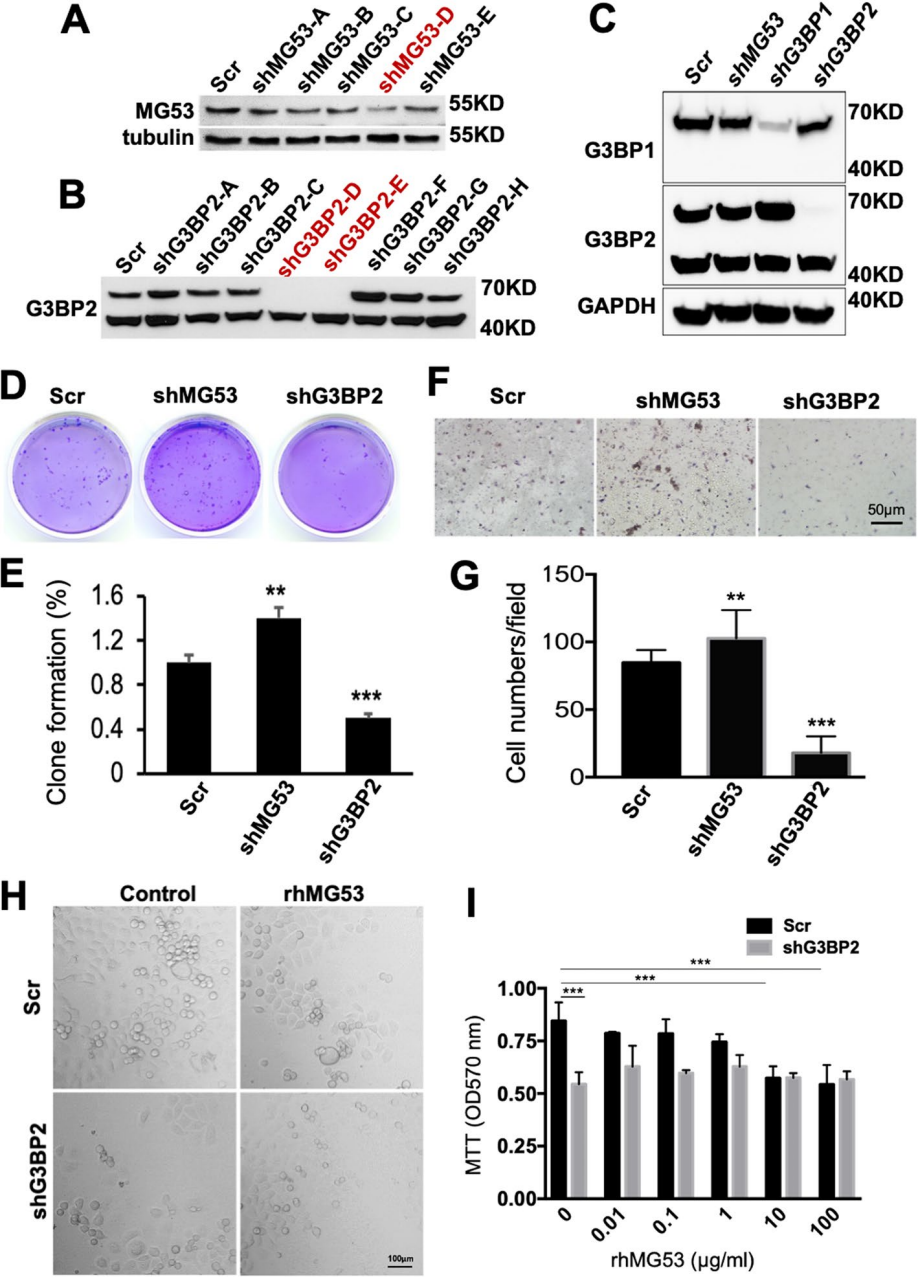
Knockdown MG53 enhances cell proliferation and migration in NSCLC cells. shRNA lentivirus was used to create stable sh-scramble control (Scr), shMG53, and shG3BP2 knockdown H2122 cells. The whole cell lysates were loaded for western blot and probed for (**A**) MG53 and (**B**) G3BP2 expression. Tubulin served as a loading control. **C** Expression G3BP1 and G3BP2 in Scr control, shMG53, shG3BP1, and shG3BP2 cell lines. GAPDH serves as loading control. H2122 cells with stable knock-down of MG53 or G3BP2 or the control cells expressing scramble constructs were performed with colony formation assay and (**D**) trans-well assay (**F**). Quantification of clone formation (**E**) and trans-well assay (**G**). ** P<0.01 and *** P<0.005 for the indicated group. **H** Representative images of Scr control and shG3BP2 cells with 72-hour incubation of BSA (control) or rhMG53 (20 μg/ml). **I** MTT assays of Scr control and shG3BP2 cells reveal different dose-dependent effect of rhMG53 on cell proliferation. * indicate significant difference with *p*<0.001. Data represents 3 independent experiments

We used shRNA to knock-down the expression of MG53 and G3BP2 and establish stable cell lines of H2122 cells with a down-regulation of MG53 (shMG53) or G3BP2 (shG3BP2). Western blot assay confirmed the reduced expression of MG53 in shMG53 cells (Fig. 4A), and reduced expression of G3BP2 in shG3BP2 cells (Fig. 4B). Consistent with the result shown in Supplemental Figure S2A, where similar levels of G3BP2 were detected in lung tissues derived from WT and *mg53*^*-/-*^ mice, there were no detectable changes in the expression of G3BP2 in H2122 cells with knockdown of MG53 (Fig. 4C). Colony formation assay was performed to compare the proliferation of shMG53 and shG3BP2 cells with the control cells (Scr, treated with a non-specific shRNA). As shown in Fig. 4D, shMG53 cells demonstrated enhanced cell growth capacity compared with control cells. In contrast, shG3BP2 cells showed significantly reduced proliferation compared with Scr cells (Fig. 4D and E).

To determine whether knockdown of MG53 and G3BP2 affected cell migration and invasion, we performed a matrigel-based transwell assay. The cell migration capacity was enhanced by ~20% in sh-MG53 cells and reduced by ~60% in shG3BP2 cells, compared with the control cells (Fig. 4F and G).

Using MTT assay, we compared the dose-dependent effect of rhMG53 on the proliferation of Scr control and shG3BP2 cells (Fig. 4H and I). As expected, the shG3BP2 cells showed ~40% slower proliferation than the Scr control cells at 72 hours post-seeding in the 96-well plate when rhMG53 was absent from the culture medium (MTT reading of 0.844±0.086, Scr control; 0.544±0.055, shG3BP2, p<0.001, 2-way ANOVA). In a dose-dependent manner, rhMG53 present in the culture medium produced anti-proliferative effect on Scr control cells, with significant reduction of MTT observed at 10 μg/ml and 100 μg/ml rhMG53 (p<0.001). Interestingly, such anti-proliferative effect of rhMG53 was completely lost with the shG3BP2 cells. These findings suggest that rhMG53 can inhibit proliferation of human NSCLC cells, and such effect is likely mediated by G3BP2.

### rhMG53 reduces arsenic trioxide (ATO)-induced SG formation in human NSCLC cells

**Fig. 5.**
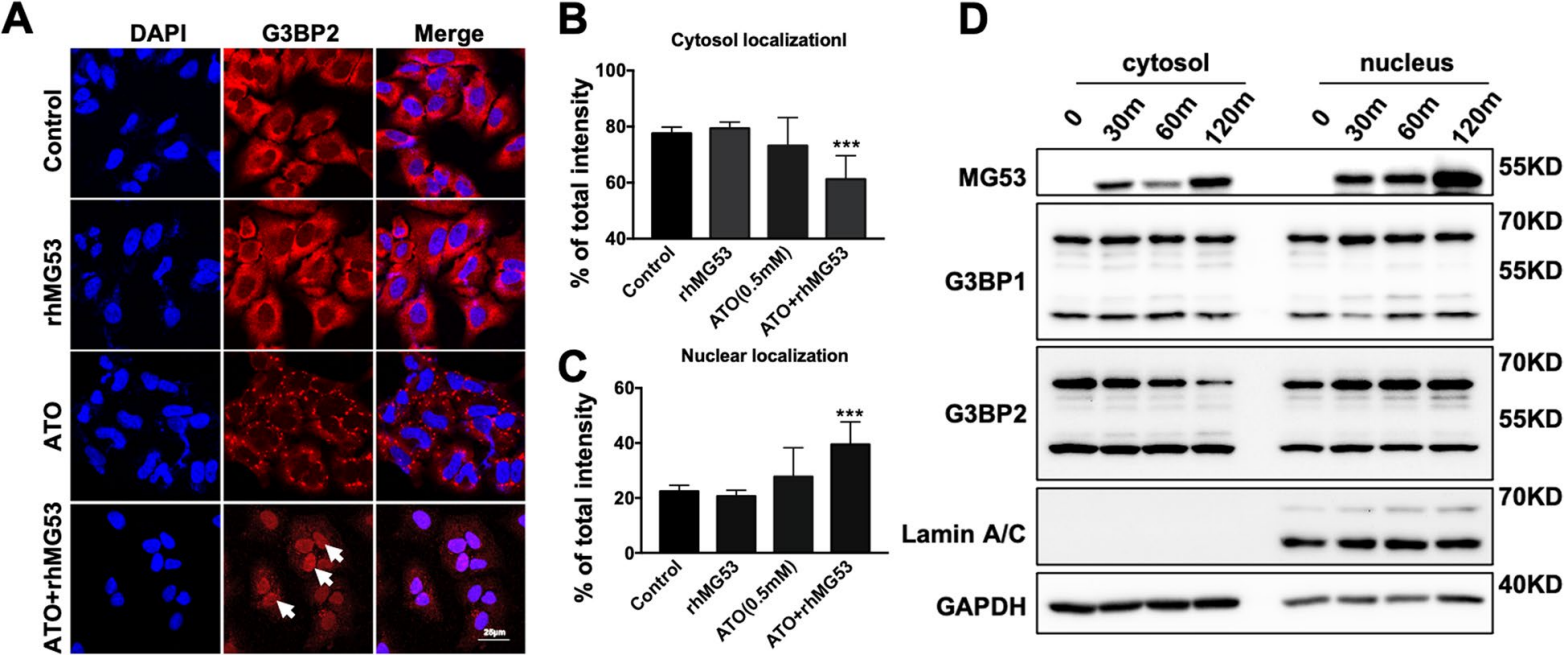
rhMG53 promotes G3BP2 nuclear translocation and reduces SG formation in A549 cells sunder ATO-induced stress conditions. **A** A549 cells were treated with rhMG53 (10 μg/mL), ATO (0.5 mM), or ATO (0.5 mM) plus rhMG53 rhMG53 (10 μg/mL) for 40 min, and the distribution of G3BP2 was analyzed by IHC staining under confocal microscopy. The nucleus was stained with DAPI (blue). Arrows indicate G3BP2 nuclear localization. Quantifications of the (**B**) cytosolic and (**C**) nuclear aggregation of G3BP2. *** *P*<0.005 for the indicated group. Data represents 3 independent experiments. **D** A549 cells were treated with ATO (0.5 mM) plus rhMG53 rhMG53 (10 μg/mL) (lower panels) for 0, 15min, 60min, and 120 min respectively. The cytoplasmic and nuclear protein extraction were subjected to western blot analysis with anti-G3BP1 and G3BP2 antibodies. GAPDH and Lamin A/C served as loading controls

Using ATO-induced oxidative stress model [58], A549 cells were treated with 0.5 mM ATO for 40 min. As shown in Fig. 5, ATO treatment led to puncta SG formation of G3BP2 in the cytosol. The number and intensity of ATO-induced SGs in A549 cells were reduced when rhMG53 was added to the culture medium (ATO+rhMG53, 10 ug/ml). Close examination revealed that rhMG53 treatment induced nuclear localization of G3BP2 (marked by arrows in the bottom right panel with DAPI co-staining, Fig. 5A), which was rarely seen without rhMG53 (ATO alone). Quantification from multiple experiments indicated that rhMG53 significantly suppressed SG formation in the cytosol (Fig. 5B) and induced nuclear translocation of G3BP2 (Fig. 5C). rhMG53 induced nuclear translocation of G3BP2 was further confirmed using subcellular fraction assays. The western blots shown in Fig. 5D demonstrated that the time-dependent accumulation of G3BP2 in the nuclear fraction was enhanced in A549 cells after treatment with ATO and rhMG53.

G3BP1 and PABP-1 are functional and spatially linked to SGs [14, 15, 59]. To further confirm the function of MG53 in the suppression of the SG formation, SGs were induced in A549 and H460 cells by ATO treatment, and co-staining of G3BP2 and G3BP1 were performed with A549 cells (Supplemental Figure S3A), as well as G3BP2 and PABP-1 (poly-A binding protein 1) with H460 cells (Supplemental Figure S3B). Clearly, ATO-induced SG formation was suppressed by rhMG53 in both H460 and A549 cells, which were both associated with nuclear accumulation of G3BP2.

### rhMG53 enhances cisplatin-induced cell death in A549 cells

**Fig. 6:**
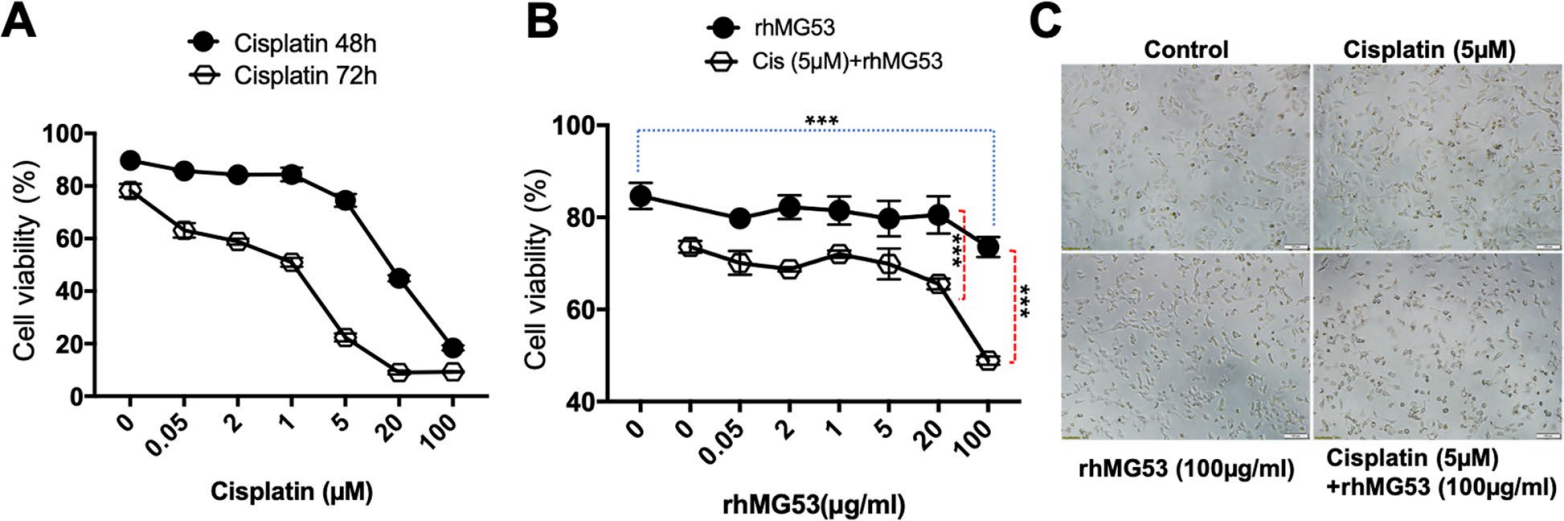
rhMG53 enhances cisplatin-induced cell death in A549 cells. **A** Dose-dependent changes in A549 cell viability were established with incubation of cisplatin for 48 hours (*closed circle*) or 72 hours (*open circle*). **B** A549 cells were cultured in the presence of rhMG53 or rhMG53 plus cisplatin (5μM) at indicated doses for 48 hours. Data presented are mean ± SD of four duplicates each. *** P<0.001 for the indicated group. **C** Representative images of cells treated with cisplatin (5μM), rhMG53 (100μg/ml), or combination of rhMG53 and cisplatin

Previous studies have shown that cancer cells develop resistance to chemotherapeutic treatment by the excessive formation of SGs [23–29]. We thus postulate that MG53-mediated control of SG formation may potentially enhance the sensitivity of NSCLCs to chemotherapeutics. *In vitro* assay with A549 cells was conducted to evaluate the combinatory effect of rhMG53 and cisplatin to cause death of lung cancer cells. We first conducted experiments to establish the dose-dependence of cisplatin to induce death of A549 cells by incubating the cells with varying concentrations of cisplatin for 48 hours or 72 hours (Fig. 6A). As expected, longer incubation for 72 hours caused a leftward shift in the dose-response relationship of cisplatin to induce death of A549 cells, compared to those observed with 48 hours incubation. When varying concentrations of rhMG53 (0.05 to 20 μg/ml) were added to the culture medium, there were minimal changes in viability of A549 cells. Significant changes in cell viability was only measured with 100 μg/ml rhMG53 following 48 hours incubation (84.6±2.8% control vs 73.6±2.2%, +100 μg/ml rhMG53, *p* = 0.008) (Fig. 6B, filled circles). Importantly, while incubation of A549 cells with 5 μM cisplatin alone for 48 hours only caused a small reduction in cell viability, the presence of rhMG53 appeared to augment the cytotoxic effects of cisplatin. With the combination of 20 μg/ml rhMG53 and 5 μM cisplatin, cell viability was reduced to 65.5±0.5%, compared to 73.6±1.3% (cisplatin alone). Moreover, with the combination of 100 μg/ml rhMG53 and 5 μM cisplatin, cell viability was further reduced to 48.9±0.9%. Representative images of A549 cells treated with 48 hours incubation of cisplatin (5 μM), rhMG53 (100 μg/ml), or combination of cisplatin and rhMG53 are shown in Fig. 6C. These data reveal the synergistic effect of rhMG53 and cisplatin to induce A549 cell death.

### Overexpression of MG53 suppresses tumorigenesis in vivo

**Fig. 7:**
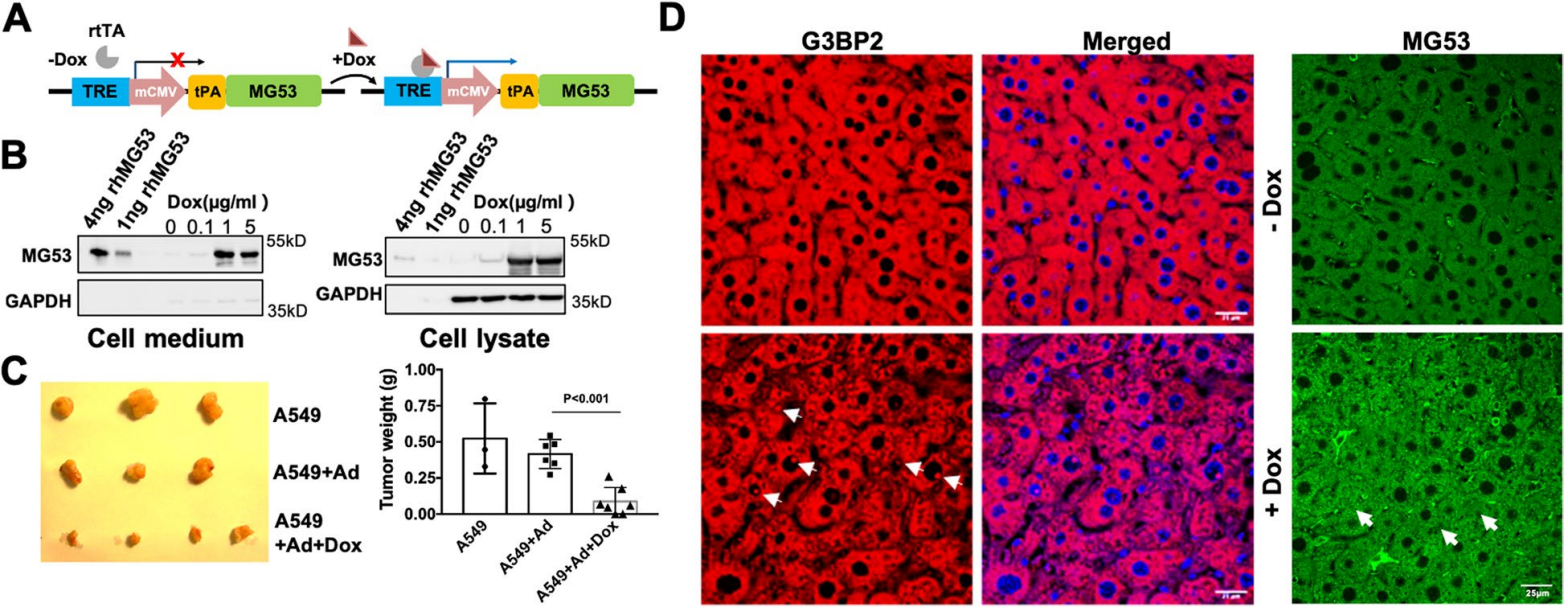
Overexpression of MG53 suppresses A549 xenograft tumor growth *in vivo*. **A** Schematic representation of the Ad-tPA-MG53 cDNA under the control of a tetracycline response element (TRE) [60], allowing to tailor the expression and secretion of MG53 in a Dox-inducible manner. **B** A549 cells were infected with adenovirus (*Ad-TRE-tPA-MG53*), induced with or without Dox for 24 hr, then cells and conditioned culture medium were harvested and analyzed. Western blot analysis revealed that with addition of Dox (0.1, 1, and 5 μg/ml), dose-dependent elevation of MG53 (*right panel*) with concurrent secretion of MG53 into the culture medium was observed. GAPDH served as a loading control. **C** A549 xenograft weight from mice treated with Dox were significantly smaller than those from animals treated with the control saline group or non-Dox treatment group (*n*>6 for each group). **D** Primary tumors from mice were subjected to IHC staining with anti-G3BP2 antibody. The nucleus was stained with DAPI (blue). Arrowheads indicate the nuclear localization of G3BP2. **E** IHC staining with MG53 showed increased levels of MG53 with Dox-treatment (*bottom panel*). Arrowheads indicate nuclear localization of MG53. Because antibodies against G3BP2 and MG53 used for the immunostaining were all derived from rabbits, separate IHCs with G3BP2 and MG53 were conducted with different sections of the xenograft

We developed a novel approach with doxycycline (Dox)-inducible secretion of MG53 from lung cancer cells (Fig. 7A). A tissue-plasminogen activator (tPA) sequence was added to the 5’ end of the human MG53 cDNA to enable the secretion of MG53 into the medium. The tPA-MG53 transgene, which was used for the generation of the tPA-MG53 mice [41], was cloned behind a mini-CMV promoter controlled by the tetracycline response element (TRE) [60], allowing for tailored induction of MG53 expression and secretion by doxycycline (Dox). The TRE-tPA-MG53 construct was packaged into the adenovirus for high-efficiency infection of human lung epithelial cells.

As shown in Fig. 7B, in the absence of Dox, A549 cells infected with Ad-TRE-tPA-MG53 did not show overexpression or secretion of MG53, confirming the tight control of the TRE element. With addition of Dox (0.1, 1, and 5 μg/ml), the dose-dependent elevation of MG53 (*right panel*) with the concurrent secretion of MG53 into the culture medium was observed.

We next performed an *in vivo* study with transplantation of A549 cells infected with Ad-TRE-tPA-MG53 into the flanks of the immune-deficient NCR nude mice. As shown in Fig. 7C, mice that were treated with subcutaneous injection of Dox (100 μg/site in 100μl saline) demonstrated reduced tumor growth, compared to those treated with saline control. This is associated with Dox-induced MG53 overexpression and secretion from the transplanted A549 cells. IHC staining with the explanted tumors derived from mice treated with Dox showed different patterns of SGs with more G3BP2 present in the nucleus than those derived from mice treated with saline only (Fig. 7D, *lower left panel*). Similarly, IHC staining with antibody against MG53 supported Dox-induced elevation of MG53 in the explanted xenograft; and many cells displayed nuclear-pattern of MG53 localization (Fig. 7D, *lower right panel*).

## Discussion

Since the discovery of MG53 in 2009 [33], many studies have defined the biology of MG53 in tissue repair and regenerative medicine application [34–39, 41, 49, 52, 61–71]. In addition to facilitating cell membrane repair, MG53 also has a putative tumor suppressor function [46, 47]. However, the mechanism of action of MG53 in cancer biology remains largely unknown. In this study, we identified a functional interaction between MG53 and G3BP2 for modulation of SG formation associated with lung tumor progression. Specifically, using the *mg53*^*-/-*^ mice, we found that the loss of MG53 led to the age-dependent development of lung cancer. Using allograft transplantation of mouse NSCLC cells into the *mg53*^*-/-*^, wild type, and tPA-MG53 mice, we demonstrated a physiological role of MG53 in the modulation of lung cancer growth. Knockout of MG53 leads to aggressive lung tumor growth whereas elevated levels of MG53 in circulation inhibit lung cancer tumor growth. We showed that NSCLC tissues derived from human patients contain elevated levels of G3BP2 with excessive formation of SGs. Knockdown of MG53 enhanced proliferation and migration of NSCLC cells, however knockdown of G3BP2 reduced migration and proliferation of NSCLC cells. Furthermore, the exogenous rhMG53 protein had anti-proliferative effect on human NSCLC cells, and such effect was lost with knockout of G3BP2. We conducted *in vitro* studies with multiple NSCLC cell lines, including A549, H460, and H2122 cells, and found that ATO-induced SGs could be inhibited by treatment with rhMG53. The tumor suppressor function of MG53 was further demonstrated using a xenograft model of lung cancer where inducible MG53 expression were shown to have an inhibitory role on tumor growth.

G3BP2 is an important regulator of the Ras signal pathway with oncogenic function, and also the main component of SGs. The TRIM family proteins are characterized by the conserved tripartite motif, including a RING finger, a B-box, and the coiled-coil domain [72]. Through mutagenesis analysis, we identified the TRIM domain of MG53 is involved in the physical interaction with G3BP2. Several previous studies have noted that G3BPs (G3BP1 and G3BP2) are upregulated and associated with poor prognosis in human cancers including gastric [16], breast [17, 19], lung cancer [20], sarcoma [21], prostate [22], hepatic [48], and colon cancer [73]. In this study, we confirmed that G3BP2 protein is up-regulated in human NSCLC tumor tissues. IHC staining revealed an abundant cytosolic distribution of G3BP2, which is consistent with its role in the cytosolic SG formation in human cancer patients [17, 19, 22, 56].

Cancer cells use SG formation to support survival and metastatic capacity when the cells are exposed to adverse environments [19, 23, 24, 74]. For example, most chemotherapies induce the formation of SGs and promote resistance to therapies, which are related to poor patient outcomes [26, 27, 32, 75–77]. It has been suggested that interfering with SGs’ formation may represent a potential and effective means to enhance the therapy of human cancers [19, 21, 25, 30–32]. Supporting this notion, we performed a xenograft study with A549 cells infected with Ad-TRE-tPA-MG53 to induce the overexpression of MG53 in immune-deficient mice. We found that the inducible elevation of MG53 in A549 cells inhibited the growth of solid tumors. IHC staining with the explanted tumors showed altered patterns of SGs with more G3BP2 present in the nucleus when MG53 protein is elevated. This finding is consistent with the *in vitro* study with cultured NSCLC cells, where rhMG53 treatment led to more nuclear translocation of G3BP2. Our *in vitro* study with cultured A549 cells support the potential synergy between MG53 and cisplatin to induce death of lung cancer cells. It is conceivable that a combination of rhMG53 with the conventional chemotherapeutic reagents can increase the effectiveness of these drugs to treat late-stage or drug-resistant NSCLCs. More *in vivo* studies are required to evaluate the potential combinatory effect of rhMG53 and cisplatin to determine their ability to inhibit tumor growth of drug-resistant NSCLCs, or other aggressive cancers with abnormal function of G3BP2/SG.

We have done biochemical studies to show that MG53 did not control the protein level of G3BP2 in NSCLCs. Yet, MG53 has profound effect in modulating the nuclear translocation of G3BP2 under stress conditions. Many studies in cancer biology also demonstrate that modulation of the cytosol/nuclear compartmentalization of proteins with oncogenic or tumor suppressor functions have significant impact on tumorigenesis [78]. For example, it is well known that molecular or therapeutic means to control p53 nuclear translocation represent potential effective means to treat aggressive cancers [79, 80]. Likewise, we propose that interventions to control the nuclear translocation of MG53/G3BP2 may enhance the effectiveness of chemotherapy or radiotherapy to treat drug resistant NSCLCs.

MG53, as a muscle-derived protein that circulates in the bloodstream, is known to facilitate repair of injury to multiple vital organs [81, 82]. The tumor suppressor function of MG53 has just started to be appreciated. G3BP1 is a close relative of G3BP2, and both are thought to play an important role in SGs formations. In this study, we focused on G3BP2, since our biochemical study only identifies physical interaction between MG53 and G3BP2, but not G3BP1 (data not shown). However, it is known that G3BP2 can form a hetero-multimer complex with G3BP1 during SG assembly [14, 15]. Future studies will be required to dissect the potential role of MG53 in modulating the G3BP2/G3BP1 complex formation associated with SG signaling in cancer cells.

## Conclusion

In summary, data from the current study characterized a novel function of MG53 in the modulation of G3BP2-mediated SGs associated with lung cancer tumorigenesis. rhMG53 protein can potentially be used as an adjuvant to enhance the therapy of NSCLCs. Since the TRIM domain of MG53 contains the potential active motif(s) to facilitate interaction with G3BP2, targeting the TRIM/G3BP2 interaction can potentially be explored as alternative means to treat lung cancers.

## Supplementary Information

**Additional file 1: Supplementary Figure S1**. Human NSCLC patients show elevated both G3BP1 and G3BP2 expression. The whole protein extracts from the additional 10 human NSCLC tumor were subjected to western blot analysis with anti-G3BP1 and G3BP2 antibodies compared with matching non-tumor lung tissues. Actin served as a loading control. Western blot shows elevated levels of both G3BP1 and G3BP2 in human NSCLC cancer tissues (**T**) compared to adjacent non-tumor lung tissues (**N**). The multiple bands of G3BP2 detected in the western blot may reflect the different splice variants of G3BP2 that are present in the human lung tissue.

**Additional file 2: Supplementary Figure S2**. Knockout of MG53 results in elevated stress granule formation in *mg53*^*-/-*^ lung. (**A**) The whole protein extracts from wild type and *mg53*^*-/-*^ lung were subjected to western blot analysis with anti-MG53 and G3BP2 antibodies. GAPDH served as a loading control. (**B**) Representative images of IHC staining of G3BP2 (red) from WT and *mg53*^*-/-*^ mice lungs. The nucleus was stained with DAPI (blue). Arrows indicate stress granules.

**Additional file 3: Supplementary Figure S3**. rhMG53 treatment suppresses SG formation and enhances G3BP2 nuclear translocation in multiple NSCLC cells. SGs were induced by ATO treatment. (**A**) A549 cells were treated with control (top panels), rhMG53 (10 μg/mL) (2^nd^ panels), ATO (0.5 mM) (3^rd^ panels), or ATO (0.5 mM) plus rhMG53 rhMG53 (10 μg/mL) (lower panels) for 40 min, and the cells were analyzed by IHC staining with anti-G3BP1 and anti-G3BP2. G3BP1 was used as a SG marker. The nucleus was stained with DAPI (blue). Arrows indicate G3BP2 nuclear localization. (**B**) H460 cells were treated with control (top panels), rhMG53 (10 μg/mL) (2^nd^ panels), ATO (0.5 mM) (3^rd^ panels), or ATO (0.5 mM) plus rhMG53 rhMG53 (10 μg/mL) (lower panels) for 40 min, and the cells were analyzed by IHC staining with anti-PABP-1 and anti-G3BP2. PABP-1 was used as SG marker. The nucleus was stained with DAPI (blue). Arrows indicate G3BP2 nuclear localization.

**Additional file 4: Supplementary Movie**. A549 cells were transfected with G3BP2-GFP and MG53-RFP. After 24 hr of transfection, the culture media was changed to fresh media containing Hoechst 33342 and ATO (0.5 mM) was added at the beginning of the live cell imaging. Serial live cell images were taken using Nikon A1R laser microscope (at 5 min interval). Representative cell images before ATO addition (basal) or at 30 min after ATO treatment were presented in Fig. 3C.

## Abbreviations

ATCC: American Type Culture Collection
ATO: Arsenic Trioxide
CRISPR: Clusters of Regularly Interspaced Short Palindromic Repeats
cDNA: Complementary DNA
DMEM: Dulbecco’s Modified Eagle’s Medium
Dox: Doxycycline
FBS: Fetal Bovine Serum
GFP: Green Fluorescent Protein
G3BP1: Ras GTPase-activating protein-binding protein 1
G3BP2: Ras GTPase-activating protein-binding protein 2
HA: Hemagglutinin
IACUC: Institutional Animal Care and Use Committee
IHC: Immunohistochemistry
IRB: Institutional Review Board
KO: Knock-out
MG53: Mitsugumin 53, also known as TRIM72
NIH: National Institute of Health
NSCLC: Non-small Cell Lung Cancer
OSU: Ohio State University
OSUCCC: The Ohio State University Comprehensive Cancer Center
PABP-1: Poly(A)-binding Protein 1
PBS: Dulbecco's Phosphate Buffered Saline
PCR: Polymerase Chain Reaction
PFA: Paraformaldehyde
RFP: Red Fluorescent Protein
rhMG53: Recombinant human MG53 (protein)
shRNA: A short hairpin RNA or small hairpin RNA
SGs: Stress Granules
tPA: Tissue Plasminogen Activator
TRE: Tatetracycline (tet)-responsive element
TRIM: Tripartite Motif
WT: Wild Type
