# Supplementary figures and images for: MG53 suppresses tumor progression and stress granule formation by modulating G3BP2 activity in non-small cell lung cancer
Source: Mol Cancer. 2021 Sep 14;20:118. doi: 10.1186/s12943-021-01418-3 (PMC8439062; doi:10.1186/s12943-021-01418-3)

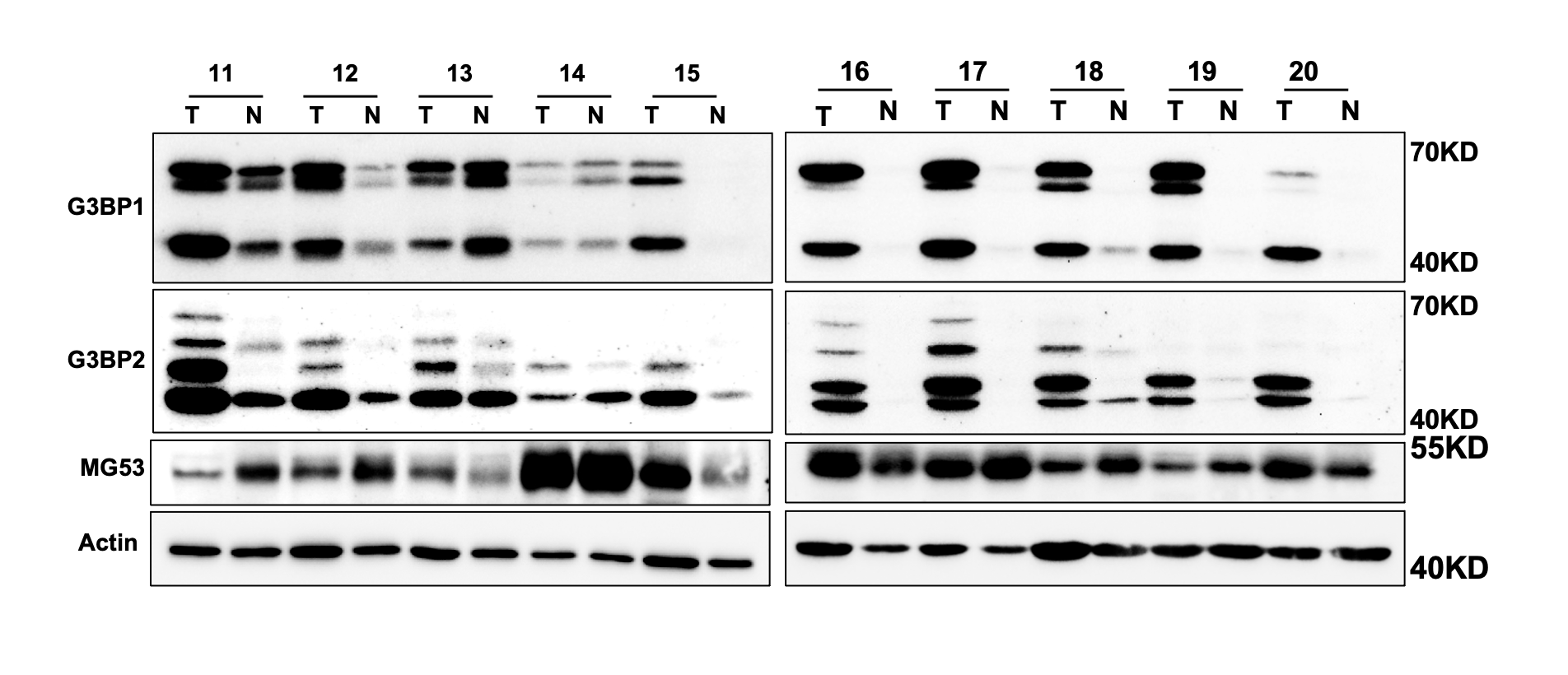

Supplement: Supplementary file 1 — Additional file 1: Supplementary Figure S1. Human NSCLC patients show elevated both G3BP1 and G3BP2 expression. The whole protein extracts from the additional 10 human NSCLC tumor were subjected to western blot analysis with anti-G3BP1 and G3BP2 antibodies compared with matching non-tumor lung tissues. Actin served as a loading control. Western blot shows elevated levels of both G3BP1 and G3BP2 in human NSCLC cancer tissues (T) compared to adjacent non-tumor lung tissues (N). The multiple bands of G3BP2 detected in the western blot may reflect the different splice variants of G3BP2 that are present in the human lung tissue. [file 12943_2021_1418_MOESM1_ESM.png]

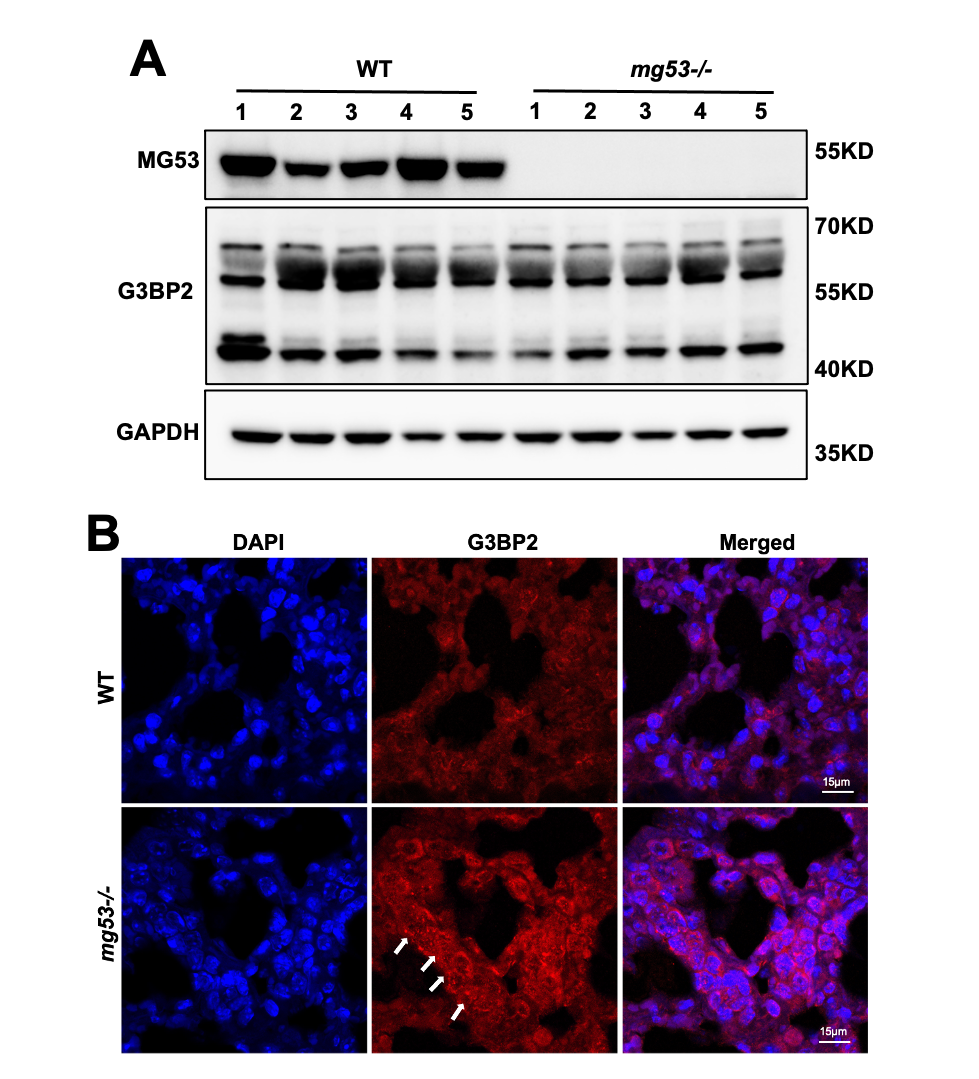

Supplement: Supplementary file 2 — Additional file 2: Supplementary Figure S2. Knockout of MG53 results in elevated stress granule formation in mg53-/- lung. (A) The whole protein extracts from wild type and mg53-/- lung were subjected to western blot analysis with anti-MG53 and G3BP2 antibodies. GAPDH served as a loading control. (B) Representative images of IHC staining of G3BP2 (red) from WT and mg53-/- mice lungs. The nucleus was stained with DAPI (blue). Arrows indicate stress granules. [file 12943_2021_1418_MOESM2_ESM.png]

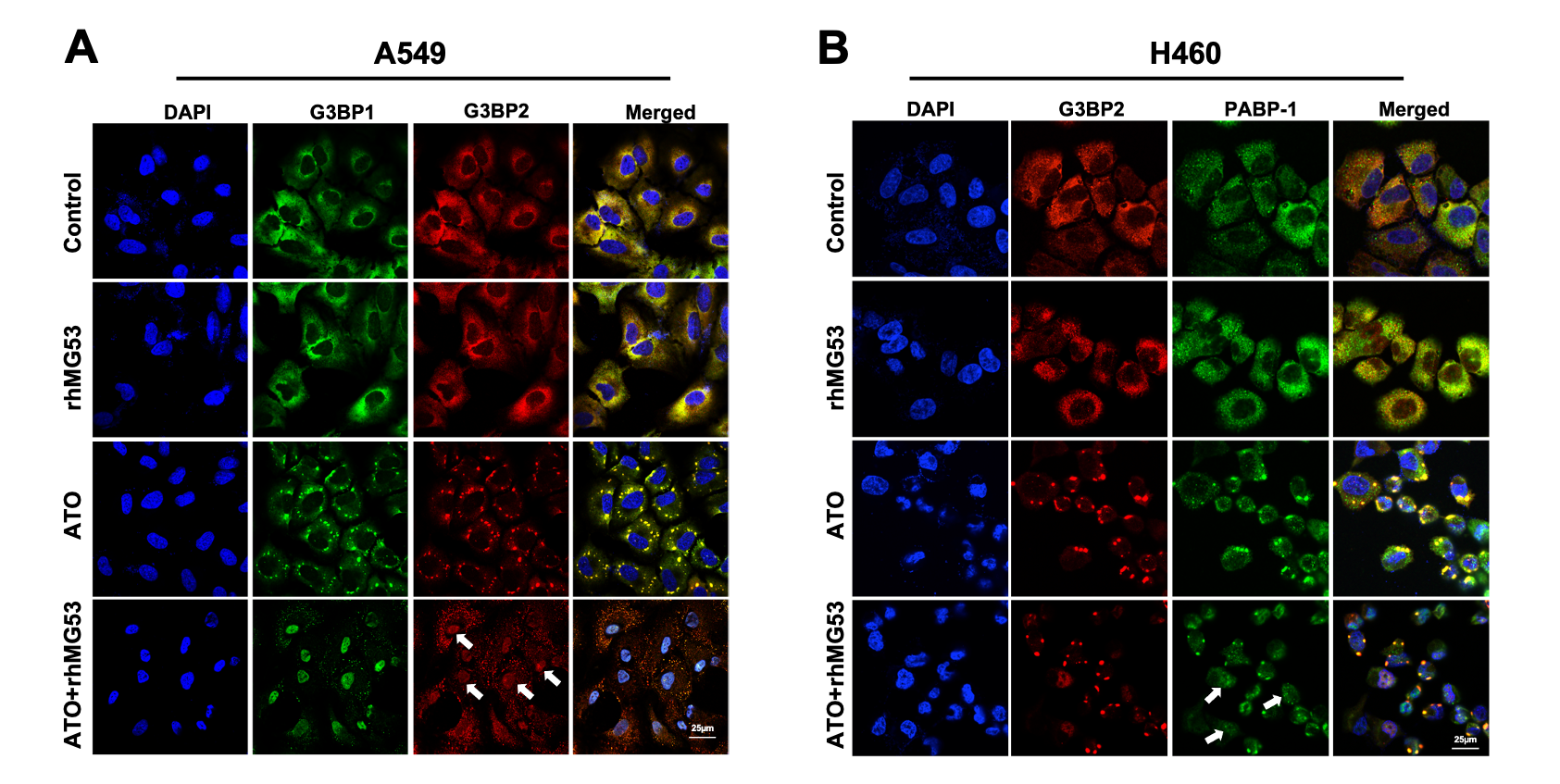

Supplement: Supplementary file 3 — Additional file 3: Supplementary Figure S3. rhMG53 treatment suppresses SG formation and enhances G3BP2 nuclear translocation in multiple NSCLC cells. SGs were induced by ATO treatment. (A) A549 cells were treated with control (top panels), rhMG53 (10 μg/mL) (2nd panels), ATO (0.5 mM) (3rd panels), or ATO (0.5 mM) plus rhMG53 rhMG53 (10 μg/mL) (lower panels) for 40 min, and the cells were analyzed by IHC staining with anti-G3BP1 and anti-G3BP2. G3BP1 was used as a SG marker. The nucleus was stained with DAPI (blue). Arrows indicate G3BP2 nuclear localization. (B) H460 cells were treated with control (top panels), rhMG53 (10 μg/mL) (2nd panels), ATO (0.5 mM) (3rd panels), or ATO (0.5 mM) plus rhMG53 rhMG53 (10 μg/mL) (lower panels) for 40 min, and the cells were analyzed by IHC staining with anti-PABP-1 and anti-G3BP2. PABP-1 was used as SG marker. The nucleus was stained with DAPI (blue). Arrows indicate G3BP2 nuclear localization. [file 12943_2021_1418_MOESM3_ESM.png]
